# A Lifeboat for Failed Nasal Reconstructions: The Supraclavicular–Submental Sandwich Flap

**DOI:** 10.1055/a-2337-2131

**Published:** 2024-08-09

**Authors:** Michel L.H.T. Vaena, Kevin Sicalo, Caterina Goulart Alessio, Eduardo Pantoja Bastos

**Affiliations:** 1 Faculdade de Ciências Médicas (FCM), Rio de Janeiro State University (UERJ), Rio de Janeiro, RJ, Brazil; 2 Hospital Universitario Pedro Ernesto, UERJ, Rio de Janeiro, RJ, Brazil

**Keywords:** surgical flaps, reconstructive surgical procedures, nose deformities, acquired

## Abstract

Many failures in total or subtotal nasal reconstruction result from an underestimation of the amount of skin required for an adequate result, especially for sufficient lining. Such planning errors usually lead to poor results, with exposure of structural grafts, infection, scar retraction, airway obstruction, and finally loss of projection and shape of the reconstructed nose. Reconstruction options for cases in which previous attempts have failed are always limited, as well as in cases of trauma or burns affecting the soft tissues of the forehead and face. In such complex situations, one may employ free flaps or tissue expansion, but such resources may not be always available. We describe a technique indicated for salvage surgeries in patients whose previous nasal reconstructions have failed, allowing a generous amount of tissue transfer for the nasal region. The technique combines the use of supraclavicular and submental flaps, with simple execution, not requiring microsurgical skills or devices such as tissue expanders. Done in three stages, the described technique provides enough skin for a total nasal reconstruction. The final result is obtained after subsequent refinements, and the total number of procedures is equivalent to when more sophisticated techniques are employed, such as tissue expansion or microsurgery.

## Introduction

Most failures in total or subtotal reconstruction of the nose come from an underestimation of the amount of tissue required for an adequate result. Such planning errors usually lead to poor results, with exposure of structural grafts and consequent infection, scar retraction, airway obstruction, and finally loss of projection and shape of the reconstructed nose. It is not uncommon for plastic surgeons to deal with unsuccessful reconstruction cases or even well-done reconstructed noses with local cancer recurrence. In these secondary or tertiary cases, as well as in cases of sequelae of trauma or burns that affect the entire face, the alternatives for reconstruction are limited and difficult. We present below a technique for salvage nasal reconstructions.

## Idea

Considering the repair of major nasal defects, the position of the nose in the midline of the face limits the reach of adjacent flaps, as these rely on terminal vascularization and therefore have limited arcs of rotation. In primary nasal reconstructions, the frontal region is the main donor area, but in larger defects of the nose (total or subtotal nasal loss) the frontal skin is insufficient to provide both coverage and lining. To address the lining defect, hinge flaps are unreliable, and septal or buccal mucosa flaps are friable and do not show the same resistance as skin flaps. In addition, factors such as previous surgeries or infections, radiation therapy, scarring, and fibrosis limit the use of local flaps. For all these reasons, total or subtotal full-thickness nasal defects, tissue expanders, or microsurgical flaps should be considered. There are some situations however, mostly in secondary or tertiary cases, in which the skin of the frontal region is no longer available for expansion, or there is no possibility for microvascular repair, either because of patient comorbidities, surgeon's inexperience, or due to health system limitations.

In such adverse situations, the “supraclavicular–submental sandwich flap” (3SF) tissue transfer technique may be used as a “lifeboat” procedure. Essentially, *it increases the coverage area and reach of the submental flap by adding an extra amount of skin from the supraclavicular flap* . Unlike old techniques such as Tagliacozzi's method or Gillies–Filatov's pedicled tubes, the 3SF technique makes use of two well-described axial flaps with robust vascularization, thus enhancing its reliability. 1 2 Besides, the main advantage of the 3SF transfer technique is that it allows full mobility of the patient's head and limbs, sparing the patient from restrictive bandages, a major drawback in the older techniques. For the reasons above, it is a useful technique for total or subtotal nasal defects, to be used as a salvage procedure when previous reconstruction attempts have failed.

### Case 1

**Fig. 1:**
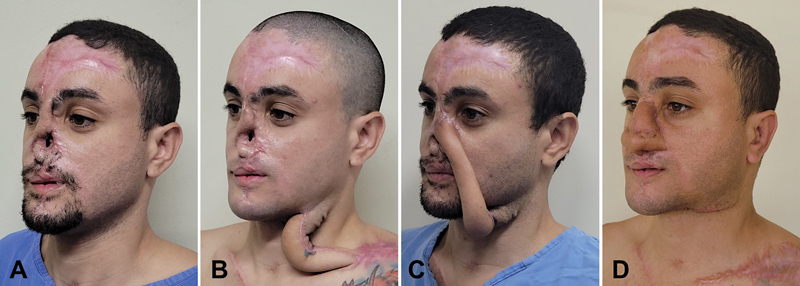
A 35-year-old man with a total nasal defect. ( **A** ) The extensive scars on the forehead denote that both paramedian flaps (right and left) were used in the previous surgeries. ( **B** ) One month after the first stage, the left supraclavicular–submental sandwich (3SF) is healed and ready to be transferred. The length redundancy of the 3SF transfer ensures no restrictions to the patient's movements. ( **C** ) After the second stage, supraclavicular extremity was sectioned in the donor area and attached to the recipient's nasal area. This attachment is done above the piriform aperture, where neovascularization will occur. ( **D** ) Final aspect after insertion of structural grafts and subsequent refinements. Both left supraclavicular and submental scars are inconspicuous.

A 35-year-old man presented with a total nasal defect. Three years earlier, the patient was involved in a motorcycle accident with major facial trauma. He underwent two previous attempts of total nasal reconstruction, having used the right paramedian forehead flap (first attempt) and a preexpanded left paramedian forehead flap (second attempt), which in turn complicated with exposure and extrusion of the costal cartilage grafts, leading to soft tissue infection and jeopardizing the entire reconstruction ( Fig. 1A ). The patient refused a microsurgical attempt (he was afraid that a radial forearm free flap would affect the functionality of his hand), so it was decided to perform a 3SF transfer technique.

#### Surgical Technique




**Video 1: Supraclavicular-submental sandwich flap – case 1**


**Fig. 2:**
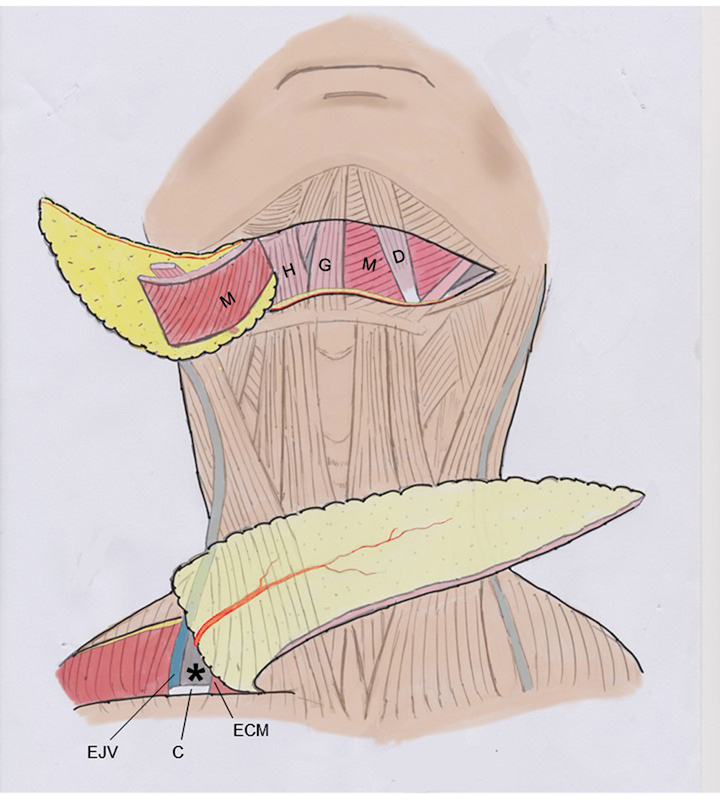
Schematic drawing of subplatysmal surgical anatomy. Above—The right submental flap is elevated together with the main portion of the right mylohyoid muscle (M). After blunt dissection of the right mylohyoid muscle (M), it can be mobilized laterally, exposing the deeper geniohyoid (G) and hyoglossus (H) muscles. The left mylohyoid muscle (M) and left anterior belly of the digastric muscle (D) remain in their anatomic positions. Below—The supraclavicular flap is raised from lateral do medial. The pedicle area is marked with an asterisk, whose anatomical landmarks can be easily identified preoperatively: the posterior edge of the sternocleidomastoid muscle (ECM), the clavicle (C), and the external jugular vein (EJV).

**Fig. 3:**
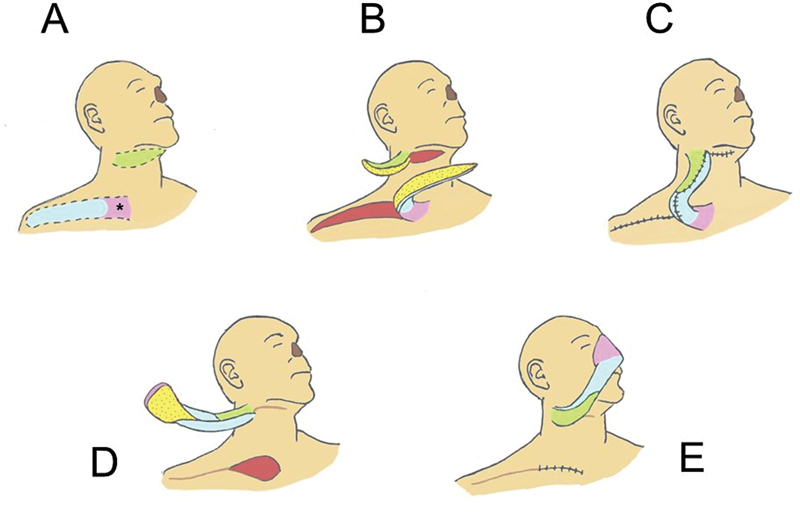
Schematic drawing of surgical stages. Above—First stage: ( **A** ) The skin paddle of the right submental flap is marked in green, and the right supraclavicular flap is marked in blue and purple. The area in purple corresponds to the skin that will cover the nose; therefore, it must have a minimum width of 7 to 8 cm. The pedicle area is marked with an asterisk. ( **B** ) Both flaps are elevated with their raw surfaces facing each other. ( **C** ) The flaps are sutured to each other, forming the “sandwich.” Note that the proximal redundant portion of the supraclavicular flap (blue area) has its raw edges sutured together forming a tube. No raw areas are left exposed. Below—Second stage: ( **D** ) After 1 month, the supraclavicular extremity is sectioned in the donor area. ( **E** ) The supraclavicular skin paddle (purple) is positioned in the recipient's nasal area. The supraclavicular donor area is closed primarily.

**Fig. 4:**
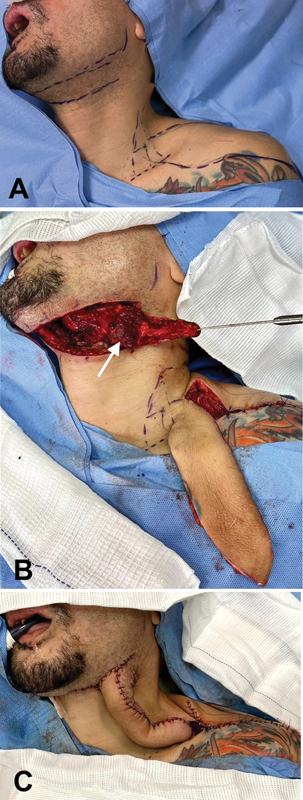
Surgical technique of the first stage in Case 1. ( **A** ) Marking for the dissection of supraclavicular and submental flaps. ( **B** ) The supraclavicular flap is raised from lateral to medial. The submental flap is dissected from medial to lateral leaving its skin portion connected to the submandibular area of the neck. The sectioned portion of the left mylohyoid muscle remains connected to the skin paddle, to avoid inadvertent injury to the submental perforating vessels (white arrow). ( **C** ) The raw sides of both flaps face one another, with the skin paddles being sutured to each other, forming a “sandwich.” Both donor areas (supraclavicular and submental) are closed primarily.

With the patient in the supine position and under general anesthesia, first, the supraclavicular flap is dissected as described by Lamberty. 3 The dissection must be performed from lateral to medial, in a subfascial plane. The easily identifiable anatomical landmarks—a triangle between the dorsal edge of the sternocleidomastoid muscle, the clavicle, and the external jugular vein—allow safety in preserving the pedicle, which does not necessarily need to be visualized if such landmarks are respected ( Fig. 2[below] ). 4 It is important to emphasize that it is the most medial portion of the supraclavicular flap that will provide the glabrous skin for the nose, therefore it is recommended that the flap has a minimum width of 7 to 8 cm in its medial (proximal) portion ( Fig. 3A ). The elevated supraclavicular flap may be up to 30 cm in length, but 22 to 24 cm is generally enough. Second, we proceed to the dissection of the submental flap. We perform the technical variation described by Patel et al, sectioning the mylohyoid muscle, which remains connected to the anterior belly of the digastric muscle, which is mobilized along with the subcutaneous tissue and skin of the submental flap. 5 This simple maneuver avoids inadvertent injuries to the submental perforating vessels, which can be thin and difficult to dissect ( Figs. 2[above] and 4B ). We prefer to maintain the skin connection between the flap and the submandibular area, to enhance the mechanical resistance of the flap—thus increasing the safeness of its transfer in the next stage—although the submental flap is described originally as an island flap. 6 Finally, the raw sides of both flaps are facing each other, with the skin portions facing outwards, forming a “sandwich.” The skin segments are sutured together and both donor areas (supraclavicular and submental) are closed primarily, leaving penrose drains ( Figs. 3C and 4C ). After 1 month, the 3SF is healed and ready to be transferred ( Fig. 1B ). During the second stage, the supraclavicular extremity is sectioned in the donor area and attached to the nasal area, with the skin tip now pointing upwards ( Fig. 3D, E ). In total nasal defects, this attachment should be done above the piriform aperture, like the technique described by Krauss for pedicled tubes. 7 The supraclavicular donor area can be closed primarily if there is no tension, in other ways, it is grafted or left to heal secondarily. Another month has waited until neovascularization of the supraclavicular extremity occurs ( Fig. 1C ). During the third stage, the submental extremity is finally sectioned and there is enough viable skin attached to the nasal area. The transferred supraclavicular skin is thin, glabrous, and well-vascularized. The excess skin length hanging over the piriform aperture, resembling an elephant's trunk, can then be used to build the entire nose. Further surgeries are required to insert structural grafts and perform refinements, which will require a minimum of four to six additional surgical stages. The patient shown in case 1 received parietal bone grafts and conchal cartilage grafts, with subsequent refinements, adding five more surgeries until the final result ( Fig. 1D and Video 1 ).

### Case 2

**Fig. 5:**
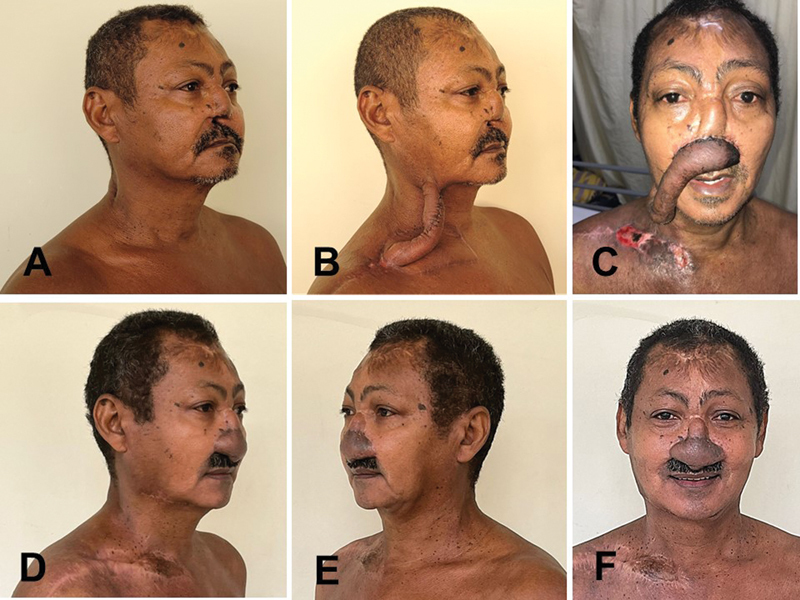
A 66-year-old man with a subtotal nasal defect. ( **A** ) Preoperative right oblique view. Previous reconstruction attempts have failed but left some frontal skin in the nasal radix and dorsum. ( **B** ) The right supraclavicular–submental sandwich flap (3SF) after the first stage. ( **C** ) Supraclavicular extremity demonstrating distal necrosis 1 week after its insertion in the nasal region. Note the length redundancy that allowed the flap to be detached, debrided, and readvanced. The wound in the right supraclavicular donor area (resulting from the second stage) can be observed. ( **D** ) Postoperative right oblique view. Supraclavicular and submental scars are unremarkable. ( **E** ) Postoperative left oblique view. ( **F** ) Postoperative frontal view. Notice the scarring on the forehead, demonstrating that both paramedian flaps (right and left) were used in the failed previous nasal reconstruction attempt.

A 66-year-old male patient presented with a subtotal nasal loss. The patient suffered major facial trauma years before, and a reconstruction attempt was performed using both left and right paramedian flaps. Both flaps presented circulatory distress and evolved with necrosis of their distal portions, leaving an incomplete nasal reconstruction, missing the nasal lobe, although some of the transferred frontal skin was left remaining in the region of the radix and nasal dorsum ( Fig. 5A ). Due to the impossibility of microsurgical treatment (patient comorbidities carried a high risk of flap failure), the 3SF technique was performed, using the right supraclavicular and submental flaps ( Fig. 5B ). As the patient did not need the skin on the radix or dorsum, during the second stage, the supraclavicular extremity was connected to the recipient area with the skin tip pointing laterally, to replace the missing nasal lobe. One week later, vascular compromise of the supraclavicular skin tip was evident ( Fig. 5C ). The flap was then detached, and the compromised segment was removed. However, the redundancy of the length of the flap allowed its reinsertion into the recipient bed and thus it was possible to proceed with the reconstruction. As seen in case 1, after neovascularization of the supraclavicular skin in the recipient region, the submental extremity was sectioned. The patient underwent four subsequent surgeries for structuring and refinements until the final result ( Fig. 5D–F ).

## Discussion

The nose occupies a unique, central position in the human face, consequently, nasal reconstruction is the archetype of all reconstructive surgeries. Because of its historical importance, and due to its impact on the patient's self-image and quality of life, nasal reconstruction has been a matter of devotion for generations of plastic surgeons. 1 Many failures in total or subtotal nasal reconstructions come from an underestimation of the amount of skin required for an adequate result, especially for sufficient lining. Such planning errors usually lead to poor results, with exposure of structural grafts and consequent infection, scar retraction, airway obstruction, and finally loss of projection and shape of the reconstructed nose. Due to such failures, the patient's situation becomes more dramatic. Because of the nose's central position, permanent social exposure of the face makes it impossible for the patient to hide the nasal deformity. At the same time, being the nose is situated in the midline of the face, the terminal vascularization reduces the options of flaps for large nasal defects.

In those complex cases where previous reconstruction attempts have failed, the fundamentals of nasal reconstruction are the same: establishing adequate lining, structure, and coverage. Structuring is usually done using costal cartilaginous grafts or bone grafts, as much in the same way as primary cases. The biggest challenge in these secondary or tertiary cases of total nasal reconstruction remains in providing the necessary lining and coverage for a satisfactory result.

If the condition of the local tissues allows it, an adequate lining can be obtained using a combination of septal and buccal mucosal flaps, along with hinged skin flaps or a folded frontal flap. When local conditions are inadequate, microsurgical flaps become necessary for adequate lining, and the radial forearm free flap should be the first choice in such circumstances, providing a generous amount of thin, pliable skin for the nasal lining. However, microsurgical flaps require a team with expertise, which may not be always available, especially in developing countries. Gasteratos et al presented a literature review that shows that the number of works published in the world is still limited and therefore microsurgical nasal reconstruction is still under development, with results dependent on experienced hands. 8 Also, comorbidities such as high blood pressure, diabetes, and smoking may limit the indication of flaps (as described above in case 2). Furthermore, it must be emphasized that microsurgical flaps may fail due to circulatory events (arterial spasm, venous thrombosis, etc.) that are independent of the surgeon's skills. Even after successful microsurgical lining, subsequent surgeries are necessary to refine the flap, adding time, cost, and emotional distress. Menick and Salibian describe a series of 38 cases using five-stage microsurgical reconstruction, of which 40% required additional surgeries. 9

To address the coverage problem, in general, a patient with a failed total nasal reconstruction should be approached using tissue expanders in the frontal region, if there is frontal skin left available for such expansion. The frontal skin has the best texture and color match for nasal reconstruction, and most forehead flaps are pedicled in the supratrochlear arteries, but one may use secondary pedicles as well (the supraorbital arteries or the frontal branch of the superficial temporal artery) according to the needs of each case. In both cases presented above, previous scarring precluded the use of these secondary pedicles. Skin expansion also brings delay and cost to the treatment, which may demand too much from a patient who is already emotionally fragile. Rezaeian et al report a successful case in which salvage surgery used three expanders concomitantly in a patient who had already been operated upon 37 times before. 10 However, often the patient with a failed previous nasal reconstruction has multiple scars in the frontal region, with the unavailability of healthy, unscarred forehead skin to be expanded. As seen in the cases presented above, the use of supraclavicular skin to cover nasal reconstruction has the disadvantage of color mismatch, which may be more apparent in dark-skinned patients. This discrepancy can be alleviated with the use of topical treatment (for example, topical hydroquinone cream) and tends to attenuate over time.

In such complex secondary or tertiary nasal reconstruction cases, where previous attempts have failed, microsurgical reconstruction may be impossible due to several reasons—lack of microsurgical team, contraindication due to patient comorbidities, poor quality recipient vessels, patient refusal, or health system limitations—such a situation may be the rule rather than the exception in developing countries. At the same time, the use of expanders may be impossible due to cost or unavailability of adequate skin for expansion. In these very adverse circumstances, the surgeon is faced with a scenario like that of the early days of plastic surgery, when both microvascular techniques and tissue expansion were not available. Surgeons at that time solved their cases using pedicled flaps. When local or regional flaps were insufficient, the amount of tissue needed for a total or subtotal nasal reconstruction was generally transferred using the so-called “Italian method,” in honor of Gaspare Tagliacozzi's technique in the 15th century. 1 Pioneer surgeons who already mastered the use of frontal flaps also transferred skin using this iconic technique, both in the second half of the 19th century and at the beginning of the 20th century. 11 Naturally, the necessary immobilization to perform the Italian method (described by Nelaton and Ombredanne as “a torture”) made this method fall into disuse with the development of the more versatile pedicled tubes by Gillies and Filatov. 2 12 Nevertheless, it should be noted that pedicled tubes also required immobilization and discomfort, but to a lesser extent. Gillies and Millard advocated the use of the acromiopectoral pedicled tube for nasal reconstruction. 13 All these older distant tissue transfers made use of random flaps, which required considerable time for their autonomization. Albeit these older techniques fell into disuse after the advent of microsurgical free flaps, medical literature shows that they are still occasionally employed. 14 15

Although the 3SF transfer technique may resemble a pedicled tube, three main differences that must be pointed out. First, the 3SF transfer uses two well-established axial flaps, thus providing greater reliability and reproducibility. Second, the contact surface between the two flaps is much greater than when a tube is applied, with a shorter time for autonomization and transfer, reducing the need for delays. Besides, in patients with unfavorable circulatory conditions, a delay may be added to increase flap safety. As shown in case 2 above, albeit vascular compromise, the long reach of the arc of rotation of the flap allowed the discard of compromised part and the reinsertion of the viable part in the recipient area. Third, the main advantage of the described technique is that it does not impose movement limitations or restrictive bracings, with more comfort during treatment.

As described, our technique does not require microsurgical skill for its execution, being done with relatively easy dissection, and making use of flaps whose anatomy is well-described and known. It does not involve the costs and time required when using tissue expanders. The 3SF technique is best suited for salvage nasal reconstruction in those patients whose previous attempts have failed, but it may be as well indicated to provide adequate lining in primary cases of total nasal reconstructions (as an option when microsurgical lining is not possible), followed by a frontal flap for coverage, when the forehead skin is available.

We have presented a technique indicated for salvage surgeries in patients whose previous nasal reconstructions have failed. Unlike methods of the past (Tagliacozzi flap or Gillies–Filatov tubes), the technique does not require immobilization of the patient. Its execution is easily reproducible and does not require microsurgical skills or tissue expanders. The technique involves tissue transfer done in three stages with subsequent refinements, and the result is obtained in several surgeries equivalent to when more sophisticated techniques (such as tissue expansion or microsurgery) are employed.
